# Tumor-promoting myeloid cells in the pathogenesis of human oncoviruses: potential targets for immunotherapy

**DOI:** 10.1186/s12935-022-02727-3

**Published:** 2022-10-27

**Authors:** Azin Aghamajidi, Pooya Farhangnia, Salar Pashangzadeh, Amirmasoud Rayati Damavandi, Reza Jafari

**Affiliations:** 1 Department of Immunology, School of Medicine, Iran University of Medical Sciences, Tehran, Iran; 2 Iranian Research Center for HIV/AIDS, Iranian Institute for Reduction of High-Risk Behaviors, Tehran University of Medical Sciences, Tehran, Iran; 3 Students’ Scientific Research Center, Exceptional Talents Development Center, Tehran University of Medical Sciences, Tehran, Iran; 4 School of Public Health, Tehran University of Medical Sciences, Tehran, Iran; 5 Cellular and Molecular Research Center, Cellular and Molecular Medicine Institute, Urmia University of Medical Sciences, Urmia, Iran

**Keywords:** Myeloid cells, Oncoviruses, Tumor microenvironment, Cancer progression, Immunotherapy

## Abstract

Oncoviruses, known as cancer-causing viruses, are typically involved in cancer progression by inhibiting tumor suppressor pathways and uncontrolled cell division. Myeloid cells are the most frequent populations recruited to the tumor microenvironment (TME) and play a critical role in cancer development and metastasis of malignant tumors. Tumor-infiltrating myeloid cells, including tumor-associated macrophages (TAMs), myeloid-derived suppressor cells (MDSCs), tumor-associated dendritic cells (TADCs), and tumor-associated neutrophils (TANs) exert different states from anti-tumorigenic to pro-tumorigenic phenotypes in TME. Although their role in the anti-tumorigenic state is well introduced, their opposing roles, pro-tumorigenic activities, such as anti-inflammatory cytokine and reactive oxygen species (ROS) production, should not be ignored since they result in inflammation, tumor progression, angiogenesis, and evasion. Since the blockade of these cells had promising results against cancer progression, their inhibition might be helpful in various cancer immunotherapies. This review highlights the promoting role of tumor-associated myeloid cells (TAMCs) in the pathophysiology of human virus tumorigenesis.

## Background

Human oncogenic viruses, known as oncoviruses, potentially contribute to an estimated 12–20% of human cancers, accounting for a large fraction of the global cancer burden [1]. Recently, several oncoviruses with DNA or RNA genomes such as human papillomavirus (HPV), Epstein-Barr virus (EBV), hepatitis B virus (HBV), hepatitis C virus (HCV), human herpesvirus-8 (HHV-8), human T-cell lymphotropic virus-1 (HTLV-1), and Merkel cell polyomavirus (MCV) have been recognized as the primary contributors to cancer development [2]. Viral carcinogenesis is a complex process associated with viral factors and immune escape mechanisms. There is interesting crosstalk between different viral and host factors which mediate the signaling pathways and cellular process. In general, oncoviruses can inhibit the tumor suppressor pathway p53, which supports primary tumor growth and progression [3]. It has also been demonstrated that viral factors potentially activate PI3K-Akt-mTOR, Notch, and Wnt pathways leading to cell overgrowth, tumor invasion, and angiogenesis [4]. On the other hand, oncoviruses establish an infection-associated chronic inflammation that could mediate cancer development through different mechanisms including tissue remodeling, angiogenesis, and production of growth factors [5]. The tumor microenvironment (TME) consists of different immune cells which play a prominent role in the tumor progression. Myeloid cells are the heterogeneous population of the innate immune system, which is considered the first line of defense. These cells include tumor-associated macrophages (TAMs), myeloid-derived suppressor cells (MDSCs), tumor-associated dendritic cells (TADCs), and tumor-associated neutrophils (TANs) that predominantly infiltrate the TME [6]. Despite the central role of myeloid cells in regulating anti-tumor immune responses, tumor-associated myeloid cells (TAMCs) can promote tumorigenesis mechanisms [7]. It is noteworthy that TAMCs exert crucial pro-tumorigenic functions in regulating cancer-related inflammation, expression of pro-angiogenic factors, tumor angiogenesis, tumor progression, and promotion of immune evasion [8]. The pro-tumorigenic functions of TAMCs, including anti-inflammatory cytokine secretion and chronic ROS production, have been considered significant obstacles to developing effective cancer treatments. Therefore, TAMCs are considered a double-edged sword of immune effectors in cancer progression. Given the dual role of TAMCs in cancer development and their therapeutic potential, this review highlights the role of tumor-promoting myeloid cells in the pathogenesis of human oncoviruses and provides new insights into cancer immunotherapy.

## Anti- and pro-tumorigenic function of myeloid cells in cancer pathogenesis

Myeloid cells exert an immunosuppressive activity to combat the proliferating tumor cells; however, it has been demonstrated that they represent opposing functions from anti-tumorigenic to pro-tumorigenic phenotypes in the TME. Hence, we briefly describe the mechanism of the anti- and pro-tumorigenic function of TAMCs in the immune escape and cancer pathogenesis.

### Tumor-associated regulatory dendritic cells (TAR-DCs)

Dendritic cells (DCs) are a double-edged sword population in the TME. Plasmacytoid (pDC), conventional (cDC1 or cDC2), and inflammatory DC (moDC) are three phenotypically and functionally distinct subsets of DCs [9]. These immune cells play a crucial role in various cancer types, including breast, lung, colorectal, ovarian, head and neck, bladder, gastric, and renal cancer [10]. Although, DCs mediate antigen trafficking and stimulation of CD8^+^ T-cell responses, however, TAR-DCs exhibited immunosuppressive properties by low expression of costimulatory molecules and high expression of regulatory molecules. Stromal-cell derived factor-1 (SDF-1) which is also known as CXCL12, in the TME of malignant tumors and high expression of CXCL4 ligand results in the accumulation of DCs in TME. Immunoglobulin-like transcript 7 (ILT7) recognizes bone marrow stromal cell antigen 2 (BST2), which is highly expressed on tumor cells, resulting in negative regulation of the interferon responses [11]. It has been demonstrated that IL-10 produced by TAMs potentially suppresses the secretion of IL-12, which mediates immune escape and metastatic progression. The inhibition of IL-10 could restore the functionality and cytokine production of DCs [12].

### Tumor-associated macrophages (TAMs)

Tumor-associated macrophages (TAMs) are abundant myeloid cells in the TMEwith anti-tumorigenic or strongly pro-tumorigenic phenotypes. Macrophage colony-stimulating factor (M-CSF) is highly expressed in the TME, which recruits the macrophages from the bone marrow or spleen [13]. TAMs are classified as classically activated-M1 and alternatively activated-M2 macrophages which induce anti-tumorigenic Th1 immune responses and pro-tumorigenic functions such as tumor growth and invasion, immune suppression, and, angiogenesis which is mediated by cytokine and chemokine production respectively [14]. M1 macrophages exert anti-tumor activity by direct cytotoxic effects mediated by ROS production, and antibody-dependent cell-mediated cytotoxicity (ADCC) to eliminate tumor cells [15]. M2 macrophages are predominantly the vast majority of non-malignant TAMs associated with the production of immunosuppressive chemokines and factors including TGF-β and IL-10. Furthermore, TAMs are related to angiogenesis by producing pro-angiogenic factors, including vascular endothelial growth factor (VEGF), fibroblast growth factor (FGF), platelet-derived growth factor (PDGF), and matrix metalloproteinase (MMP) [13]. TAM can enhance tumor proliferation and invasion mediated by activation of NF-κB and STAT3 and expression of pro-inflammatory cytokines [16]. Elevated levels of TAMs are correlated with poor prognosis of diverse types of cancers [17, 18].

### Myeloid-derived suppressor cells (MDSCs)

MDSCs are developmentally immature non-macrophage cells with an immunosuppressive function. These cells potentially prevent the activation of CD4^+^ and CD8^+^ T-cells. Also, it has been suggested that MDSCs suppress NK cells, which may disturb anti-tumor immunity [19]. Therefore, MDSCs are considered a serious hurdle against cancer immunotherapy. There are distinct subsets of MDSCs that express heterogeneous markers, including Siglec-3/CD33, CD14, CD15, and CD66b. However, CD11b is expressed by all types of human MDSCs. MDSCs exert immunosuppressive function through the production of IL-10, TGF-β, ARG1, IDO, and CD40 [12]. MDSCs inhibit T lymphocytes via the ROS or the depletion of L-arginine (L-arg) [20]. MDSCs suppress NK cells by expressing transforming growth factor β (TGF-β) and decreasing the expression of the NK-cell activating receptor NKp30 [21]. MDSCs also inhibit myeloid cell differentiation via a ROS-dependent mechanism [22].

### Tumor-associated neutrophils (TANs)

As the first line of immune defense, neutrophils are a substantial population that infiltrates the TME. TANs have a dual function of anti- and pro-tumor activities, modulating anti-tumor immunity [23]. Interestingly, TANs are classified as two major types, N1 and N2, with anti-tumor and pro-tumor functions, respectively [23]. N1 mediates direct and indirect anti-tumor activity by ROS production and H2O2 and ADCC that could effectively kill tumor cells [24]. TANs actively contribute to tumor proliferation, angiogenesis, tumor progression, and metastasis through the high-level expression of neutrophil elastase and matrix metalloproteinase 9 (MMP9) [25]. Moreover, upregulation of TANs in the TME strongly predicts the poor survival rate in patients with cancer [26]. TANs could modulate innate and adaptive immune responses by different mechanisms. As an instance, they decrease the CTL response by upregulation of arginase-1. Furthermore, the production of neutrophil-secreted neutrophil elastase (NE) leads to tumor cellular proliferation. TANs mediate angiogenesis by secretion of VEGF and hepatocyte growth factor (HGF) [23].

### Tumor-promoting myeloid cells and human oncoviral infection

Here we highlight the mechanistic strategies by oncoviral infection in immune disturbances (Table 1).

**Table 1:** Key mechanisms employed by oncoviruses

Virus	Oncogene/Oncoprotein Signaling pathway	Downstream consequence
Epstein-Barr virus (EBV)	LMP1	Upregulation of IL-1, IL-6, and GM-CSF MDSC proliferation Increased M2 macrophages Inhibition of NK cell and T-cell
Hepatitis B virus (HBV)	HBx	Increased M2 macrophages Activation of NKG2D in NK cells T cell senescence
Hepatitis C virus (HCV)	Core protein	Activation of TLR2/PI3K/AKT/STAT3 signaling cascade Inhibition of CD4 + T cells MDSC proliferation Downregulation of IFN-γ
Human herpesvirus 8 (HHV-8)	vFLIP	Induction of CD11b + Gr1 + cells MDSC proliferation
Human papillomavirus (HPV)	E6, E7	Inhibition of p53, MDSC proliferation Increased Treg cell Downregulation of IFN-γ

### Epstein-Barr virus (EBV)

Epstein-Barr virus (EBV), first identified in the tumor cells of Burkitt lymphoma, is now associated with a strikingly diverse variety of lymphoproliferative lesions and malignant lymphomas of B, T, and NK cell origin [27]. Here, we highlight the association between EBV and tumor-promoting myeloid cells such as MDSCs and TAMs.

#### MDSCs

The latent membrane protein-1 (LMP1) is the primary oncogene of EBV that plays a critical role in the MDSCs proliferation and tumor immunosuppression. A large fraction of MDSCs is found in patients with EBV-associated T/NK cell lymphoproliferative diseases, which may dampen the antiviral T-cell responses [28]. LMP1-mediated glycolysis enhances the production of IL-1β, IL-6, and GM-CSF, the proliferation of tumor-associated MDSCs, and the inhibition of T-cells and NK cells, which lead to tumor immunosuppression [29, 30]. The accumulation of PMN-MDSCs in nasopharyngeal cancer survivors with persistent hepatitis B may suppress the host immune response [31] to the Epstein-Barr virus and be linked to tumor recurrence via ER stress/ROS pathway.

#### TAMs

In gastric cancer, the EBV-encoded miR-BART11 targets FOXP1 to enhance the tumor-associated macrophage-induced epithelial-mesenchymal transition [32]. Zhang et al. revealed that in nasopharyngeal carcinoma (NPC) cells, EBV induced M2 phenotype in TAMs and elevated the p-ATR expression. These two inductions were highly connected and linked to higher tumor staging, lymph node metastases, and poor patient prognosis [33]. Activation of ATR triggered by EBV increased subcutaneous tumor development, elevated Ki67 production, and lung metastasis in nude mice through the M2-type TAMs recruitment [33]. CD68 as a TAMs marker was higher in EBV-positive NPC. However, between EBV-positive and EBV-negative NPC, there was no variation in M2 macrophage number [34]. The survival of EBV^+^ tumor cells is dependent on TAMs in the EBV-positive TME [35]. The EBV status of lymphoma cells affected TAMs by up-regulation of CXCR10 and VEGF, causing angiogenesis and tumor survival. The in vivo reduction of macrophages revealed that they are required to survive EBV-positive tumor cells [35].

EBV expression has been identified in lesional macrophages of different cancers, ranging from thyroid to uterine carcinoma and some types of lymphoma that possibly elicit EBV lytic infection of macrophages in many tumor-associated macrophages in EBV-related malignancies [36].

In classical Hodgkin’s lymphoma, tumor-infiltrating macrophages are linked to a poor prognosis and the presence of EBV [37]. Increasing the number of TAMs is related to a reduction in overall survival, while greater levels of markers are statistically substantially associated with the presence of EBV infection [38].

### Hepatitis B virus (HBV)

The most frequent kind of liver cancer is hepatocellular carcinoma (HCC). HBV is a chronic infection that affects over 350million individuals worldwide. At least half of all HCC cases globally are caused by chronic hepatitis B virus (HBV) infection [39]. Here, we highlight evidence of the association between HBV and tumor-promoting myeloid cells.

#### MDSCs

HCC patients had considerably greater percentages of MDSCs and PMN-MDSCs than chronic hepatitis B patients and healthy controls [40]. Pal et al. have demonstrated that the induction of regulatory T-cells (Tregs) by myeloid-derived suppressor cells in persistent HBV infections featuring high viral surface antigen is long-lasting and persists following antiviral treatment [41]. In the chronic liver failure posed by HBV, the proliferation of myeloid-derived suppressor cells was strongly associated with the severity and course of the disease [42].

#### Macrophages

Macrophages are monocytic phagocytes with antigen-presentation and cytokine-producing capabilities. The tissue-specific liver macrophages are Kupffer cells dominating other innate immune cells in the organ [43]. From the onset of HBV infection through the beginning and development of HCC, macrophages act as the key mediator of the pathogenic process. Kupffer cells have a role in inflammatory responses and tolerance generation in the early stages of infection [44]. In a specifically modified murine model of HBV infection, liver dysfunction was linked to an enormous frequency of human M2 macrophages [45]. Kupffer cells may impede the progression of HBV-associated HCC by inhibiting T-cell-mediated anti-tumor activity, limiting T-cell activation with PD-L1 expression on monocytes, and causing Tim3^+^/CD4^+^ and Tim3^+^/CD8^+^ cells to senescence [46, 47].

#### NK cells

The lymphocytes in the human liver tissue are predominantly natural killer cells (NK cells). Patients with persistent HBV and HCV infection have more NK cells in their liver [44]. A ligand of the NKG2D receptor, MICA, is upregulated in HBV infection, and soluble MICA levels have been linked to modulating responses directed by NK cells, which are essential in developing HCC in HBV-associated HCC patients [44, 48]. Several mechanisms have been shown to selectively impair NK cell function after chronic HBV infection. These include TGF-β and IL-10 stimulation of NK cells and their increased expression of Tim-3 triggered by HBV, hindering their activity [49, 50].

### Hepatitis C virus (HCV)

HCV infection affects more than 270million individuals globally. HCV produces a chronic and lifelong infection in most infected individuals. This persistent inflammation in the liver leads to macronodular cirrhosis in 20% of people who contract it. A 4 to 7% yearly risk of progressing to HCC is associated with these individuals [51].

Through the TLR2/PI3K/AKT/STAT3 signaling cascade, HCV induced MDSC-like suppressive monocytes that activated CD4^+^Foxp3^+^ Tregs and inhibited the autologous CD4^+^ T-cell activation [52]. HCV stimulates the accumulation of CD33^+^ MDSCs, which reduces T-cell responsiveness via the production of ROS [53]. IFN-γ production by natural killer cells is suppressed by MDSCs induced by HCV, which alter cellular metabolism by inhibiting arginase-1 [54]. MDSCs triggered by HCV promote the development of Tregs while inhibiting the activity of effector T-cells [55]. Hepatitis C core protein polarizes granulocytic myeloid-derived suppressor cells via the IL-10/STAT3 signaling [56].

A long non-coding RNA (lncRNA) named HOXA transcript antisense RNA myeloid-specific 1 (HOTAIRM1) targets HOXA1 gene expression to regulate myeloid cell development. HOTAIRM1 enhances MDSCs growth and suppressive activities during HCV infection through the HOXA1-miR124 axis [57]. Exosomes associated with HCV suppress miR-124, which promotes the growth of myeloid-derived suppressor cells [58]. RUNX1 overlapping RNA (RUNXOR) is another lncRNA that targets runt-related transcription factor 1 (RUNX1) and is crucial for myeloid cell development. Exosomes associated with HCV through the STAT3-miR124 axis upregulate RUNXOR and RUNX1, increasing the MDSCs population and suppressive capabilities [59].

### Human herpesvirus 8 (HHV-8)

The causative agent of Kaposi’s sarcoma (KS) is Kaposi’s sarcoma herpesvirus (KSHV; also known as human herpesvirus 8 (HHV-8)). KS is the most prevalent neoplasm among untreated HIV patients, although it may also happen in immunosuppressive conditions after organ transplantation [60]. KSHV vFLIP is a latent infection-associated viral oncoprotein. CD11b^+^Gr1^+^ cells with suppressor immune phenotype are induced by vFLIP, which remodels myeloid differentiation and causes their proliferation [61]. Based on the evidence, DC exerts decreased antiviral immune responses and altered cytokine production during the HHV-8 infection [62]. It has also been demonstrated that HHV-8 infection is associated with prostate cancer [63]. Moreover, the study on the Iranian population has indicated the high prevalent rate of the HHV-8 genome among patients with cervical cancer [64]. Therefore, it can be perceived that HHV-8 infection may be associated with an increased risk of cervical cancer.

### Human papillomavirus (HPV)

Cervical cancer is caused by certain HPV (human papillomavirus) genotypes. Other anogenital cancers and a subset of head and neck cancers seem to be caused by the same genotypes. It is necessary to sustain the malignant development of cervical cancer cells by inducing the expression of particular viral oncoproteins, E6 and E7, which specifically inhibit the tumor suppressors p53 and RB [65]. In malignancies pertaining to HPV, MDSCs are related to both poor clinical outcomes and resistance to treatment. They inhibit the activity of CTLs, downregulate IFN-γ, and increase the frequency of Tregs, all of which contribute to carcinogenesis. Furthermore, compared to normal controls, their levels were elevated, indicating a clear relationship between pathological grade and their levels [66–68]. Activating CD8^+^ effector memory T-cells and controlling MDSCs together allowed protection against cancers caused by the HPV-16 serotype [69].

**Fig. 1:**
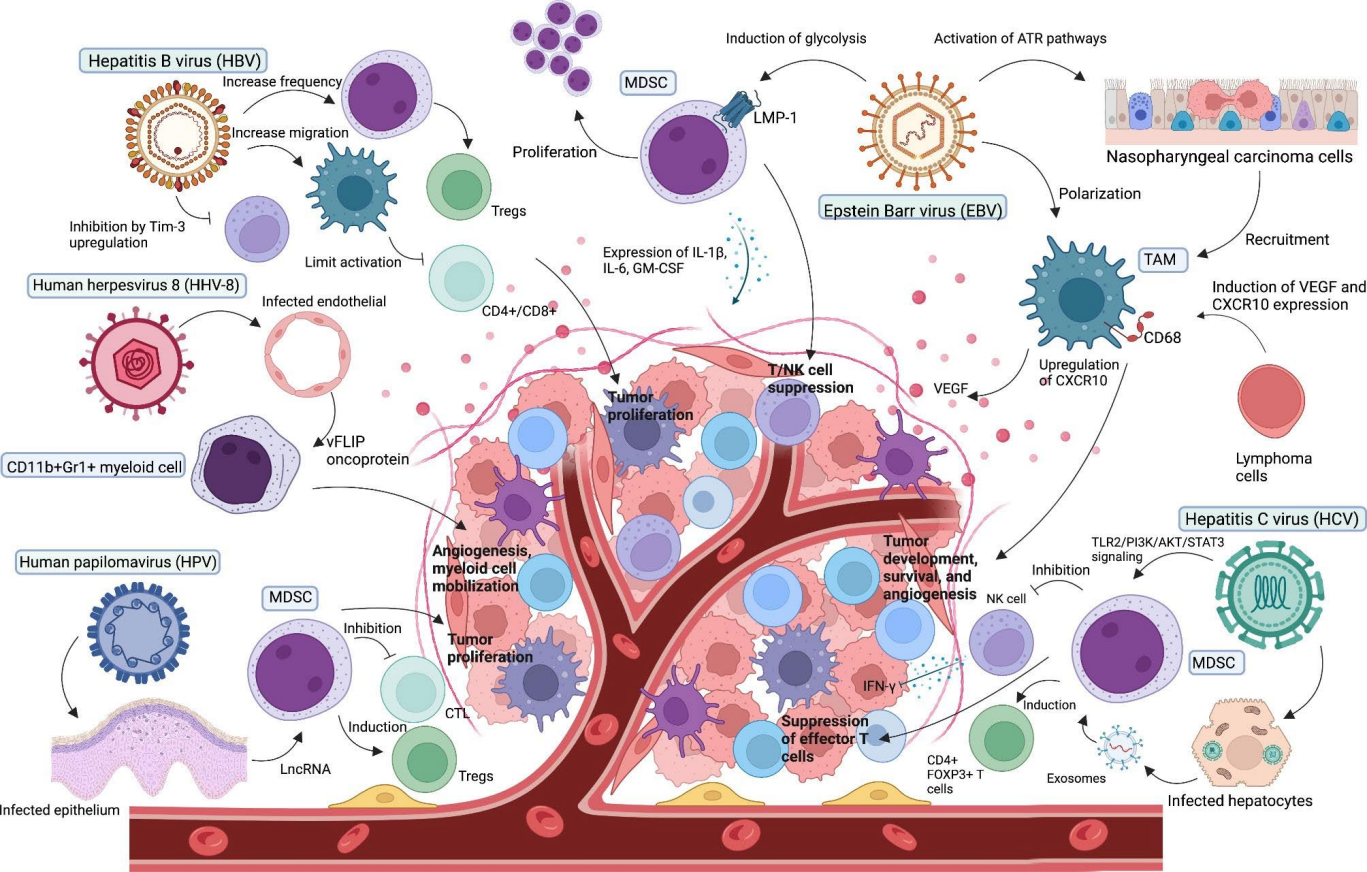
Tumor promoting myeloid cells and their role in pathogenesis of human oncoviruses. Here, five human oncoviruses (Epstein-Barr virus, Hepatitis B and C viruses, Human herpesvirus 8, and human papillomavirus) have been illustrated with their related myeloid cells mostly tumor-associated macrophages (TAMs) and myeloid-derived suppressor cells (MDSCs). These cells along with immune mediators in tumor microenvirnment promote tumor progression, angiogenesis, and migration and suppress anti-tumor effector cells including T and NK cells

The oncogenic mechanisms of tumor-promoting myeloid cells in reviewed human oncoviral infections have been depicted in Fig.1.

### Tumor-promoting myeloid cells and potential targets for immunotherapy of human oncoviruses

Due to the immunosuppressive nature of MDSCs and TAMs, these cells have been considered two main potential targets for cancer immunotherapy. Although, different therapeutic approaches to target these immunosuppressive myeloid cells are being investigated. Here, we will provide the strategies for repolarization and revival of tumor-promoting myeloid cells (Table 2).

**Table 2:** The strategies of repolarization and revival of tumor-promoting myeloid cells

Mechanism	Molecular and/or Cellular Target	Agent_(s)_	Reference
Blocking recruitment	CXCR4/CXCL12 axis	AMD3100	[125]
	PI3Kγ	IPI-549	[126]
	mTORC	Rapalogs	[127]
	BTK	Ibrutinib	[128]
	CCR2/CCL2	Carlumab, C1142, Bindarit	[129–132]
Inhibiting differentiation	CSF1R/CSF1 axis	Emactuzumab (RG7155), Cabiralizumab (FPA008), Pexidartinib, ARRY-382, SNFX-6352, BLZ945, AMG820, IMC-CS4, LY3022855	[93, [133–140]
CD40 activation	CD40	Selicrelumab, APX005M, SEA-CD40, CP-870-893	[141–[144]
TLR activation	TLR1	Pam3	[145]
	TLR2	Pam3-CSK4, SMU-Z1, LTA	[145–147]
	TLR3	Poly:IC	[148]
	TLR4	GSK1795091	[149]
	TLR6	LTA	[150]
	TLR7/8	NKTR262, Resiquimod, Imiquimod, SM-052	[151–153]
	TLR9	IMO-2125, CMP-001, SD-101 CpG	[154–156]
Immune checkpoint blockade	SIRPα/CD47 axis	CV1, TTI-621, Hu5F9-G4	[157–159]
	MARCO	mAbs (Unknown)	[160]
PI3K inhibition	PI3K	SF1126, SRX3207, Clotrimazole	[111–113]
HDAC inhibition	HDAC	TMP195	[116]
Angiogenesis inhibition	VEGF/VEGFR axis	mAb	[161]
Apoptosis	TAM	Zoledronate, Trabectedin	[82], [84]
Inhibition of ATP synthesis	ATP synthase	Oligomycin, 2-Deoxyglucose	[162], [163]

### Blocking recruitment

Blocking the recruitment of MDSCs and TAMs may be a beneficial strategy for reducing tumorigenesis and immunosuppression. CCR2^+^ TAMs and MDSCs in TME are recruited by the CCL2 chemokine [70–72]. Blocking CCL2/CCR2 axis reverses MDSCs infiltration into the tumor, augmenting the effectiveness of the cancer immunotherapy [73]. In breast cancer models, removing CCR2 blockade induces tumor progression, migration, and angiogenesis [74]. Clinical studies are now underway for anti-CCR2 agents, including carlumab (CNTO 888), PF-04136309, MLN1202, BMS-813,160, and CCX872-B [75, 76].

The CXCL12/CXCR4 axis governs TAMs’ migration into hypoxic tumor areas through the endothelial barrier [77]. Targeting the CXCL12/CXCR4 axis in multiple cancer models, including prostate and breast cancer, reduces tumor burden and metastatic susceptibility by preventing TAM infiltration [78, 79].

### Depleting macrophage populations in the TME

TAMs are among the most common and important non-neoplastic cell groups in the established TME. The differentiation of macrophages into tumor-suppressive M1 or tumor-promoting M2 types is an important stage in the formation of the TME. Implementing three strategies through this pivotal axis could pave for novel cancer treatment strategies. These strategies could alter M2 TAM survival and apoptotic mechanisms or disrupt their signaling pathways, suppress chemotactic potential toward the tumor, and reprogram M2 TAMs to produce M1 phenotype macrophages [80].

Bisphosphonates elicit myeloid cell cytotoxicity by preferentially targeting phagocytic cells, including TAMs [81]. Zoledronate, a third-generation bisphosphonate, is cytotoxic to TAMs that express matrix metalloproteinase-9 (MMP9) and improves macrophage anti-tumor activity by polarizing monocytes toward pro-inflammatory phenotype [76, 82]. Trabectedin, a drug mainly used for soft tissue malignancies, inhibits TAMs, enhancing anti-cancer adaptive immunity in response to anti-programmed cell death protein 1 (PD-1) treatment [83]. Trabectedin causes mononuclear phagocytes to undergo accelerated apoptosis. In animal tumor models, trabectedin reduced angiogenesis by selectively depleting monocytes/macrophages in the blood, spleens, and tumors [84].

### Reprogramming metabolism

Several agents, including growth factors, could modify macrophages’ immune and metabolic responses in their residing microenvironment. This mechanism is reflected in the tricarboxylic acid (TCA) cycle disruption in M1 macrophages with the stimulation of inflammatory mediators resulting in IL-1 and Fatty acid synthesis and switching to pro-inflammatory phenotype [76, 85–88]. M2 macrophages, on the other hand, have an intact TCA cycle by external anti-inflammatory stimulation, which promotes mitochondrial oxidative phosphorylation (OXPHOS), yielding a higher ATP production [76, 89]. Inhibiting ATP production in M2 macrophages with an ATP synthase or a hexokinase inhibitor decreases anti-inflammatory characteristics and suppresses pro-tumorigenic function [90, 91].

### Reprogramming cellular signaling

To induce tumoricidal potential in MDSCs and TAMs, several factors could be used to reprogram their signaling pathways, including colony-stimulating factor 1/colony-stimulating factor 1 receptor (CSF1/CSF1R) blockade, TLR agonists, PI3K inhibitors, CD40 agonists [64], and Class IIa histone deacetylase inhibitors (HDACis) [76, 92, 93]. Promising targets are the macrophage surface receptors that aid antibody-dependent cellular cytotoxicity/phagocytosis (ADCC/ADCP). Macrophages harbor a membrane protein called signal regulatory protein alpha (SIRP-α) binding to CD47 molecules expressed on tumoral cells, which help them evade tumor immunosurveillance [94]. However, anti-SIRPα antibodies cause tumor cell phagocytosis while preserving T-cells [95].

TLRs agonists could induce pro-inflammatory and anti-tumor phenotypes in TAMs. Feng et al. developed a glucomannan polysaccharide with acetyl modification to the degree of 1.8 (acGM-1.8), which stimulates TLR2 signaling and promotes macrophages toward becoming anti-tumor [96]. TLR7/8 agonist-loaded nanoparticles augment cancer immunotherapy via polarizing TAMs [97]. TLR-3 stimulation via modulating IFN-αβ signaling restricts tumor progression by skewing M2 macrophages to the M1 phenotype [98]. TLR 7/8 agonists also stimulate human MDSCs to differentiate toward anti-tumor M1-like macrophages, which may reverse the suppressive action of MDSCs [99].

CSF1/CSF1R blockade in pancreatic cancer models could enhance immune checkpoint T-cell therapy outcomes while reprogramming TAMs [100]. Moreover, blocking the CSF1/CSF1R axis reduces mesothelioma growth and improves anti-PDL1 immunotherapy efficacy [101], and CSF1R inhibition minimizes the development of cervical and mammary tumors in mice by lowering TAMs turnover and increasing the CD8^+^ T-cell infiltration [102]. Inappropriate response to immunotherapy in indoleamine 2,3-dioxygenase-expressing malignancies may be overcome by targeting MDSCs with CSF1R inhibition [103]. Pro-tumorigenic TAMs are reduced, and pro-tumorigenic PMN-MDSCs are recruited when CSF1R is inhibited [104]. Indeed, CSF1R suppression enabled tumor-infiltrating PMN-MDSCs to be recruited by carcinoma-associated fibroblasts. Thus, CXCR2 inhibitors may augment the anti-cancer effects of CSF1R inhibition by preventing PMN-MDSCs recruitment [104].

TAMs are sensitive to profound and abrupt reprogramming in the presence of a CD40 agonist when CSF-1R signaling is inhibited. Despite the short window of macrophage hyperactivation, simultaneous CSF-1R inhibition plus CD40 stimulation is adequate to establish a pro-inflammatory TME that revives an efficient immune response for T-cell immune checkpoint therapy [105]. Likewise, CD40 agonist, combined with CSF-1R, blockades reconditions TAMs and promotes potent anti-tumor immunity [106]. Activated macrophages with CD40 agonist invaded tumors immediately, were tumoricidal, and aided tumor stroma elimination [107]. In a pancreatic cancer mouse model, dendritic cell vaccination and CD40-agonist combined treatment enable T-cell-dependent anti-tumor immunotherapy [108].

A first in vivo evidence revealed that pharmacological suppression of the PI3K p110δ subunit inhibits the growth of breast cancer by specifically targeting cancer cells and macrophages [109]. Li et al. indicated that TAM accumulation in the glioblastoma microenvironment is suppressed by PI3K inhibition, which results in an extraordinary temozolomide response [110]. A pan-PI3K inhibitor (SF1126) reduced VEGF and other pro-angiogenic factors released by macrophages, blocking tumor-induced angiogenesis [111]. Joshi et al. demonstrated anti-tumor immunity by macrophage Syk-PI3Kγ axis [112]. Additionally, tumor immunosuppression is relieved by SRX3207, a novel dual Syk-PI3K inhibitor [112]. Clotrimazole has anti-cancer characteristics in a mouse melanoma model, functioning as a PI3K inhibitor and causing TAMs to repolarize [113].

HDAC inhibition with trichostatin-A increases anti-PD-L1-mediated tumor suppression and potentiates macrophage anti-tumor activity [114]. TMP195, an HDAC Class IIa inhibitor, may transform tumor-infiltrating monocytes and macrophages into cells able to sustain a robust CD8^+^ T-cell-mediated anti-tumor immune response in breast cancer and reduce metastasis [115, 116].

### Immune checkpoint blockade (ICB)

The PD-1 and cytotoxic T lymphocyte-associated protein 4 (CTLA-4) immune checkpoints are predominantly produced by effector immune cells, including T and NK cells. Targeting these molecules have exciting therapeutic potential by affecting myeloid biology [117, 118]. Because PD-L1 is expressed on MDSCs and TAMs, ICB using anti-PD-L1 may directly impact myeloid cell activities in TME [119]. There is a difference in response to PD-1 and PD-L1 inhibition in myeloid cells, with the latter leading to more potent immune responses by activating inflammasomes and expressing IL-18 [120]. The protective immune response to tumor cells requires inflammasome activation [121].

CD47SIRPα axis has been identified as a critical macrophage immune checkpoint. CD47 is a “don’t eat me” signal that is overexpressed in myeloid malignancies and causes tumors to evade macrophage phagocytosis. CD47 blockade causes leukemic cells to be engulfed and therapeutically eliminated [122]. CD47 blockade combined with trastuzumab eradicates HER2-positive breast cancer cells while also overcoming trastuzumab resistance [123]. Radioresistant breast cancer cells are eliminated when CD47 and HER2 are blocked [124].

## Conclusion

According to the clinical and pre-clinical evidence, TAMCs play a dual role in cancer via anti-tumorigenic and pro-tumorigenic effects. TAMCs have pro-tumorigenic and immunosuppressive functions by different mechanisms including TGF-β and IL-10 anti-inflammatory cytokine secretion, ROS production, and mediation of angiogenesis through VEGF and HGF production. Hence, TAMCs could be actively involved in cancer progression, and immune escape results in poor prognosis, adverse clinical outcomes, and a low response rate to cancer treatment. Although diverse cancer-related immunotherapies such as ICBs have been investigated, targeting promoting pathways orchestrated by myeloid cells could shed a light on a new therapeutic approach and may improve cancer immunotherapy. Blocking myeloid cells’ recruitment, macrophage population depletion, reprogramming of metabolism, and cellular signaling might be considered helpful strategies for repolarization and revival of tumor-promoting myeloid cells.

## Data Availability

Not applicable.

## **Abbreviations**

mAb: Monoclonal antibody.
TAM: Tumor-associated macrophage.
TLR: Toll-like receptor.
HDAC: Histone deacetylase.
VEGF: Vascular endothelial growth factor.
CSF1: Colony-stimulating factor 1.
CSF1R: Colony-stimulating factor 1 receptor.
LTA: Lipoteichoic acid.
BTK: Bruton tyrosine kinase.
SIRPα: Signal regulatory protein alpha.
